# Association of perioperative fentanyl analog exposure with risk of psychiatric disorder in non-cardiac surgery: A 10-year retrospective study in a Korean tertiary hospital

**DOI:** 10.1371/journal.pone.0338927

**Published:** 2025-12-31

**Authors:** Dong Yun Lee, Ah Ran Oh, Rae Woong Park, Jungchan Park

**Affiliations:** 1 Department of Biomedical Informatics, Ajou University School of Medicine, Suwon, Korea; 2 Department of Anesthesiology and Pain Medicine, Samsung Medical Center, Sungkyunkwan University School of Medicine, Seoul, Korea; Maulana Azad Medical College, INDIA

## Abstract

**Study objective:**

To evaluate the risk of developing psychiatric disorders within three years after non-cardiac surgery in patients exposed to fentanyl analogs versus other opioids.

**Design:**

Retrospective observational study.

**Setting:**

Postoperative period.

**Patients:**

The study included 52,640adult patients who underwent non-cardiac surgery at Samsung Medical Center, Seoul, Korea, between January 2011 and June 2019.

**Interventions:**

Patients were divided into those exposed to fentanyl analogs and those exposed to other opioids. Propensity score matching and Cox regression analysis were used to compare the incidence of psychiatric disorders between the groups.

**Measurements:**

Psychiatric outcomes, including depression, anxiety, stress-related disorders, substance use disorders, and psychotic disorders, were assessed.

**Main results:**

The study included 52,640 patients, evenly split between the fentanyl and other opioid groups. Fentanyl exposure was associated with a higher incidence of composite psychiatric outcomes (hazard ratio [HR] 1.28 [1.13–1.46]; *P *< 0.001), including depression (HR 1.25 [1.08–1.45]; *P *= 0.003) and stress-related disorders (HR 1.45 [1.02–2.04]; *P *= 0.036). Subgroup analyses indicated increased risks in males, females, patients without alcohol history, and those not undergoing emergency surgeries.

**Conclusions:**

Perioperative fentanyl use is linked to a higher risk of psychiatric disorders compared to other opioids. These findings emphasize the need for careful use and monitoring of fentanyl in surgical patients, considering both immediate and long-term mental health effects.

## Introduction

Opioids are the cornerstone in managing moderate to severe pain and are widely used for postoperative pain control even after minor surgical procedures [1,2]. However, they are well known for several side effects, ranging from acute responses such as nausea, itching, sedation, and hypotension to respiratory depression and death. Opioids also are associated with long-term psychiatric effects such as tolerance, dependence, and addiction [3]. Thus, opioid prescription necessitates careful consideration and monitoring due to the potential for these dose-dependent side effects.

Fentanyl is a synthetic opioid with a remarkable potency and rapid onset of action [4]. The faster onset of action and greater potency are crucial for perioperative pain control [5–7] but also contribute to the risk of adverse effects [7]. Faster entry into the brain could induce acute respiratory depression and also promotes addiction due to the quick rush, affecting the central nervous system [8]. While the acute effects of fentanyl analogs are well-documented, ramifications extend beyond immediate concerns, prompting questions on potential long-term side effects. A recent cohort study howed that opioid prescription on discharge after surgery was associated with adverse events in long-term follow-up.

The potential for development of psychiatric diseases is one of the concerns associated with opioid exposure, which has been linked to alterations in neurotransmitter systems, heightening vulnerability to psychiatric disorders [9]. Despite numerous studies on the association of opioids with psychiatric disorders, studies have mostly focused on chronic exposure [10,11]. Considering the neurobiological impact and narrower safety margin of fentanyl analogs compared to other opioids, the potential long-term side effects of their use during the perioperative period need to be investigated, particularly in association with psychiatric diseases [7]. In this study, we utilized a large real-world dataset and identified patients exposed to opioids during the perioperative period of non-cardiac surgery. The primary objective of this study was to evaluate the risk of developing psychiatric disorders within three years after non-cardiac surgery among patients exposed to fentanyl analogues compared with those exposed to other opioids. Psychiatric disorders were defined as a composite outcome including depression, anxiety disorder, stress-related disorder, substance use disorder, and psychotic disorder.

## Methods

### Data sources

For this retrospective study, we received approval from the Institutional Review Board of Samsung Medical Center (Approval ID: SMC 2021-06-078, 2022-05-26), which waived the requirement for informed consent due to the de-identified nature of the curated data. This study adheres to the Declaration of Helsinki and was reported according to the Strengthening the Reporting of Observational Studies in Epidemiology (STROBE) reporting guidelines [12].

We utilized data from the Samsung Medical Center-Non Cardiac Operation (SMC-NoCop) registry, comprised of adult patients aged 18 years and older who underwent non-cardiac surgery at Samsung Medical Center in Seoul, Korea, between January 2011 and June 2019. The raw data for this registry were obtained from a review of electronic medical charts using the Clinical Data Warehouse Darwin-C from Samsung Medical Center on 2024-05-13. This electronic retrieval system was designed for researchers to extract de-identified data from electronic medical records. With access to hospital records of more than 4 million patients, including more than 1.2 million laboratory findings and 300 million prescriptions, the system serves as a comprehensive resource. Mortality data external to our institution are regularly updated using the National Population Registry of the Korea National Statistical Office, employing personally designated identification numbers. Independent investigators have collected potential confounding variables from a preoperative evaluation sheet. The surgical risk was categorized into three groups—low, intermediate, and high—based on non-cardiac surgery guidelines from the European Society of Cardiology [13].

### Study population and exposure

**Fig 1:**
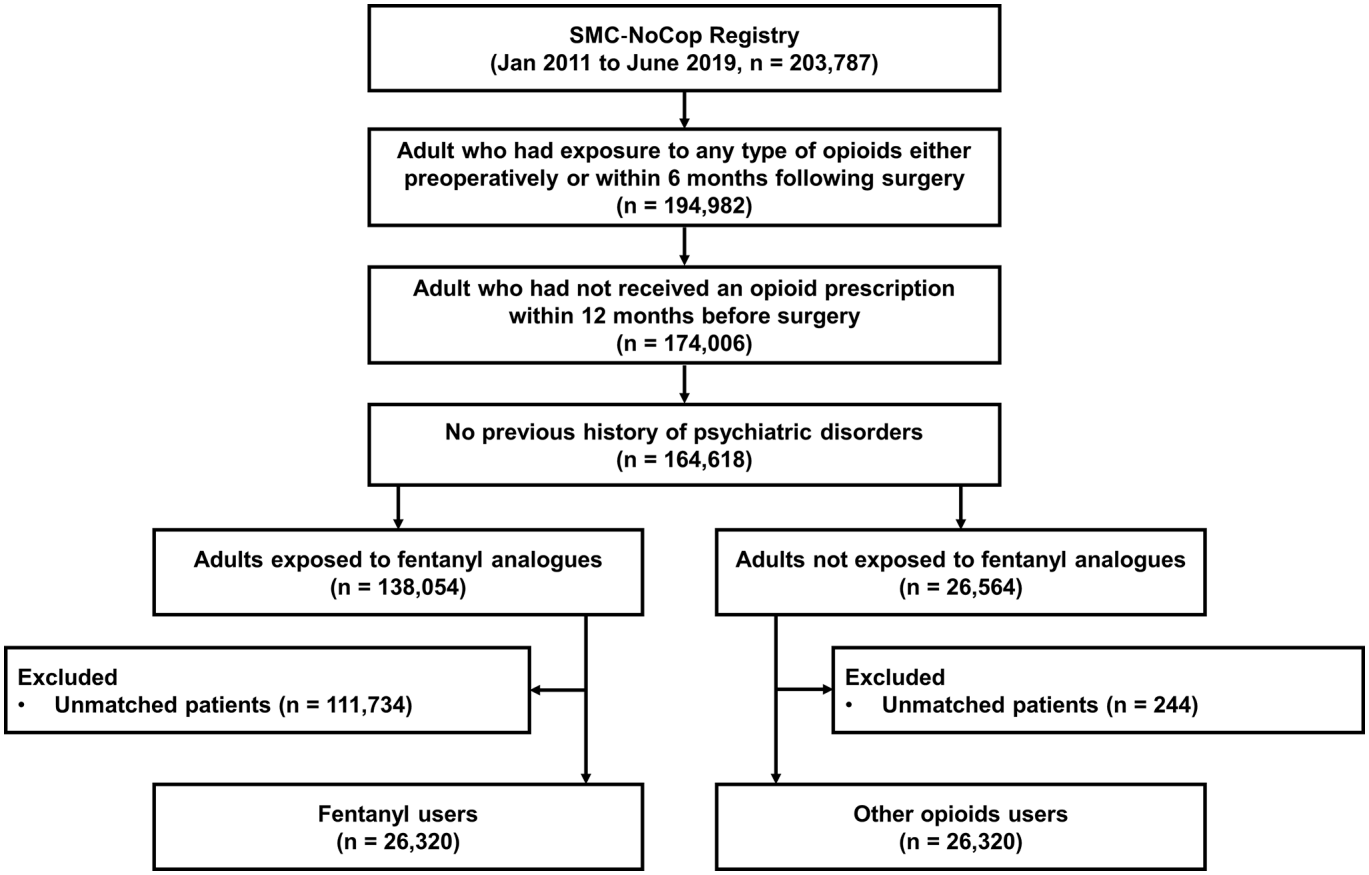
Patient selection flowchart.

For this study, we identified adults (18 years of age or older) exposed to any type of opioid either pre- or intraoperatively or within 6 months following surgery. The types of opioids other than fentanyl analogues were predominantly pethidine and morphine. For patients who underwent multiple surgeries during the observation period, was set as the index surgery. Patients who had received an opioid prescription within 12 months before the index surgery were excluded. Based on a review of existing electronic medical records, patients with documented pre-existing psychiatric disorders before surgery were excluded. No additional patient contact was performed for this study. We then stratified the remaining patients into two groups: those exposed to fentanyl analogues and those who were not. Fentanyl analogues were predominantly remifentanil (92.2%) and included fentanyl (17.4%) and sufentanil (0.01%). The use of other fentanyl analogues was extremely rare in our center and therefore not included in the analysis. The utilization of opioids at our institution lacked a standardized protocol and was entirely at the discretion of the attending clinician. A schematic visualization of the study population is presented in Fig 1.

### Outcomes and follow-up

The primary outcome was the incidence of psychiatric disorders, such as depression, anxiety disorder, stress-related disorder, substance use disorder, and psychotic disorder, within a three-year follow-up period after surgery. We also used a composite outcome of psychiatric disorders as the primary outcome, defined as the presence of any of the aforementioned psychiatric disorders. The diagnosis of psychiatric disorders was determined using the International Classification of Diseases-10.

The index date was defined as the date of surgery. The follow-up duration was calculated from the date of surgery until occurrence of the psychiatric disorder, mortality, or follow-up loss, whichever came first. Psychiatric disorders were assessed based on specific diagnostic criteria from the department of psychiatry.

### Statistical analysis

Mean with standard deviation for baseline characteristics of each user group are presented for continuous variables, and frequency with percentage are presented for categorical variables. A 1:1 ratio propensity score (PS) matching analysis was performed to adjust the effects of bias from confounders between the two user groups [14]. A PS was calculated for each patient based on a multivariable logistic regression model. The variables in the model included sex, age, body mass index, current alcohol use, current smoking status, hypertension, diabetes, chronic kidney disease, Charlson comorbidity index, stroke, coronary artery disease, arrhythmia, European Society of Cardiology/European Society of Anaesthesiology intermediate‐to‐high surgical risk, the American Society of Anesthesiologists (ASA) Physical Status Classification, emergency operation, operation duration, general anesthesia, and operation type. Operation types are presented in Table 1.

**Table 1:** Baseline characteristics before and after 1:1 propensity score matching.

	Before propensity score matching			After propensity score matching		
Characteristics	Fentanyl (*n *= 138,054)	Other opioids (*n *= 26,564)	SMD	Fentanyl (*n *= 26,320)	Other opioids (*n *= 26,320)	SMD
**Socio-demographics**						
Age, mean (SD)	52.0 (14.9)	53.4 (15.7)	0.10	54.2 (15.3)	53.3 (15.7)	0.06
Male sex, No. (%)	58604 (42.5)	12130 (45.7)	0.07	12752 (48.4)	12069 (45.9)	0.05
BMI, mean (SD)	24.1 (3.6)	24.3 (3.6)	0.07	24.3 (3.6)	24.3 (3.6)	<0.01
Smoker, No. (%)	10635 (7.7)	2370 (8.9)	0.05	2296 (8.7)	2362 (9.0)	<0.01
Use of alcohol, No. (%)	28410 (20.6)	5619 (21.2)	0.01	5369 (20.4)	5586 (21.2)	0.02
**Comorbidity**						
CCI score ≥2, No (%)	7513 (5.4)	1651 (6.2)	0.03	1768 (6.7)	1633 (6.2)	0.02
Hypertension, No. (%)	33320 (24.1)	7037 (26.5)	0.06	7432 (28.2)	6913 (26.3)	0.05
Diabetes, No. (%)	14957 (10.8)	2944 (11.1)	0.01	3383 (12.9)	2913 (11.1)	0.06
Renal failure, No. (%)	1714 (1.2)	359 (1.4)	0.01	458 (1.7)	359 (1.4)	0.03
Cerebrovascular disease, No. (%)	2540 (1.8)	392 (1.5)	0.03	559 (2.1)	392 (1.5)	0.05
Coronary artery disease, No. (%)	2459 (1.8)	447 (1.7)	0.01	629 (2.4)	443 (1.7)	0.05
Arrhythmia, No. (%)	1729 (1.3)	296 (1.1)	0.01	391 (1.5)	296 (1.1)	0.03
**Factors related to operation**						
Operation type, No (%)						
Neurosurgery	9791 (7.1)	1358 (5.1)	0.08	1140 (4.3)	1358 (5.2)	0.03
Thoracic surgery	10127 (7.3)	282 (1.1)	0.24	967 (3.7)	281 (1.1)	0.10
Head and neck surgery	23100 (16.7)	1676 (6.3)	0.28	1913 (7.3)	1676 (6.4)	0.02
Breast/endoscopic surgery	14030 (10.2)	1428 (5.4)	0.16	1667 (6.3)	1428 (5.4)	0.03
Stomach surgery	8216 (6.0)	3398 (12.8)	0.29	3112 (11.8)	3398 (12.9)	0.05
Hepatobiliary surgery	11551 (8.4)	2562 (9.6)	0.05	2526 (9.6)	2562 (9.7)	<0.01
Colorectal surgery	9017 (6.5)	2805 (10.6)	0.16	2763 (10.5)	2804 (10.7)	0.03
Urogenital surgery	10598 (7.7)	3414 (12.9)	0.19	2985(11.3)	3377 (12.8)	0.06
Gynecological surgery	18265 (13.2)	1442 (5.4)	0.23	1479 (5.6)	1442 (5.5)	<0.01
Orthopedic surgery	23359 (16.9)	8199 (30.9)	0.37	7768 (29.5)	7993 (30.4)	0.02
ASA score, No (%)						
I	62938 (45.6)	12181 (45.9)	0.01	10743 (40.8)	12142 (46.1)	0.11
II	68076 (49.3)	13271 (50.0)	0.01	14039 (53.3)	13069 (49.7)	0.07
III	6620 (4.8)	1071 (4.0)	0.04	1450 (5.5)	1068 (4.1)	0.07
IV	403 (0.3)	41 (0.2)	0.03	71 (0.3)	41 (0.2)	0.02
V	17 (0.0)	0 (0.0)	0.01	17 (0.1)	0 (0.0)	0.06
Surgical risk, No (%)^a^						
0	48451 (35.1)	11987 (45.1)	0.21	11120 (42.2)	11747 (44.6)	0.05
1	80270 (58.1)	13299 (50.1)	0.16	13593 (51.6)	13295 (50.5)	0.02
2	9333 (6.8)	1278 (4.8)	0.08	1607 (6.1)	1278 (4.9)	0.05
General anesthesia, No. (%)	128020 (92.7)	19323 (72.7)	0.77	20245 (76.9)	19322 (73.4)	0.14
Emergency operation, No. (%)	8601 (6.2)	1940 (7.3)	0.04	2031 (7.7)	1939 (7.4)	0.01
Duration of operation, mean (SD)	141.5 (103.4)	114.6 (79.9)	0.26	130.8 (102.4)	114.9 (80.1)	0.15

^a^Surgical risk was categorized according to the ESC/ESA guidelines.

SD, standard deviation; SMD, standardized mean difference; BMI, body mass index (calculated as weight in kilograms divided by height in meters squared); CCI, Charlson comorbidity index; ASA, American Society of Anesthesiologists.

Matching was performed using nearest-neighbor matching without replacement. Covariate balance between groups was assessed using the absolute standardized mean difference (aSMD), with a threshold of < 0.25 indicating acceptable balance. [15]. For all outcomes, the incidence rates (IR) per 10,000 person-year were estimated. After propensity‐score matching, we conducted Cox regression analysis to compare the incidence of psychiatric disorders. The proportional hazards assumption was assessed using Schoenfeld residuals. In the adjusted Cox models, we included postoperative fentanyl patch use (maximum dose, total amount, and timing) and additional surgery as covariates. Time-to-event outcomes were analyzed using Cox proportional hazards regression models to estimate hazard ratios (HRs) and 95% confidence intervals (CIs).

The Kaplan–Meier curve and Log-rank test were used to compare between-group differences using the log-rank test. A two-sided P value < 0.05 was considered statistically significant. Subgroup analyses were performed to determine whether the results differed according to sex, alcohol history, emergency operation, and operation type. For subgroup analyses, Benjamini–Hochberg false discovery rate (FDR) correction was conducted to account for multiplicity in multiple comparisons. The significance level was set at 0.05 using the FDR method [16]. Analyses were performed with R 4.1.0 (Vienna, Austria; http://www.R‐project.org/).

## Results

### Cohort characteristics

This analysis covered 52,640 patients, 26,320 in each group (Fig 1). The baseline characteristics of the overall study population before and after PS matching are reported in Table 1. After PS matching, all baseline characteristics were balanced between 27 matched pairs for fentanyl and other opioid users (all aSMD < 0.25; Table 1). The proportion of males in these groups was 48.4% and 45.9%, respectively. The mean age in these groups was 54.2 and 53.3, respectively. In both groups, the major operation type was orthopedic surgery (29.5% and 30.4%, respectively). In both groups, the majority of ASA scores was I or II, at 94.1% and 95.8%, respectively. Surgical risk was mostly 0 or 1 in both groups. The mean duration of operation was 130.8 minutes and 114.9 minutes in the fentanyl user and other opioid user groups, respectively.

### Outcome assessment

**Fig 2:**
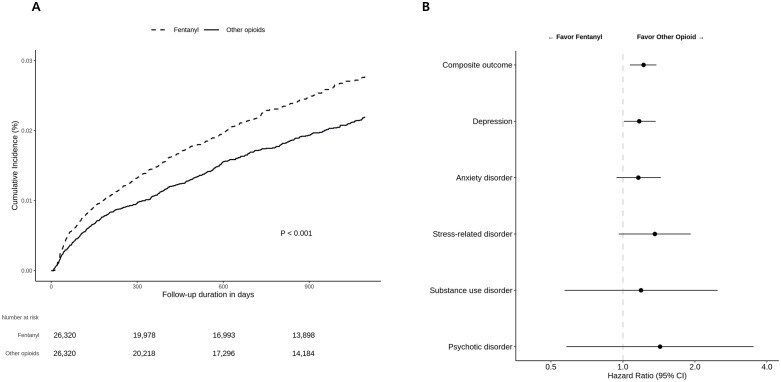
Comparison of Psychiatric Outcomes Between Fentanyl Users and Other Opioid Users. **(a)** Kaplan-Meier Plot for the Risk of Composite Outcome Between Fentanyl Users and Other Opioid Users and **(b)** Forest Plots for the Risk of Composite Outcome, Depression, Anxiety disorder, Stress-related disorder, and Substance use disorder between Fentanyl Users and Other Opioid Users.

There were significant differences in the composite outcome (IR 102.77/10 000PYs, 79.74/10 000 PYs, respectively; HR 1.28 [1.13–1.46]; *P < *0.001), depression (IR 72.31/10 000PYs, 57.67/10 000 PYs; HR 1.25 [1.08–1.45]; *P = *0.003), and stress-related disorder (IR 14.74/10 000PYs, 10.16/10 000 PYs; HR 1.45 [1.02–2.04]; *P = *0.036) between the fentanyl and other opioid user groups (Table 2; Fig 2). This significance remained consistent except for stress-related disorder after adjustment for additional surgery and fentanyl patch use (composite outcome: HR 1.22 [1.07–1.38], *P = *0.003; depression: HR 1.17 [1.01–1.37], *P = *0.037; stress-related disorder: HR 1.36 [0.96–1.92], *P = *0.086). However, the risks of anxiety disorder, substance use disorder, and psychotic disorder were not significantly different between the fentanyl and other opioid user groups.

**Table 2:** Risks of depression, anxiety disorder, stress-related disorder, and substance use disorder between fentanyl users and other opioid users.

		Hazard ratio (95% CI)	
Outcomes	Incidence Rate^a^	Unadjusted	Adjusted^b^
Composite outcome			
Fentanyl	102.77	1.28 [1.13–1.46]^*^	1.22 [1.07–1.38]^*^
Other Opioid	79.74	1 [Reference]	1 [Reference]
Depression			
Fentanyl	72.31	1.25 [1.08–1.45]^*^	1.17 [1.01–1.37]^*^
Other Opioid	57.67	1 [Reference]	1 [Reference]
Anxiety disorder			
Fentanyl	35.58	1.20 [0.98–1.49]	1.16 [0.94–1.44]
Other Opioid	29.47	1 [Reference]	1 [Reference]
Stress-related disorder			
Fentanyl	14.74	1.45 [1.02–2.04]^*^	1.36 [0.96–1.92]
Other Opioid	10.16	1 [Reference]	1 [Reference]
Substance use disorder			
Fentanyl	2.98	1.24 [0.60–2.58]	1.19 [0.57–2.49]
Other Opioid	2.40	1 [Reference]	1 [Reference]
Psychotic Disorder			
Fentanyl	2.23	1.51 [0.62–3.70]	1.43 [0.58–3.52]
Other Opioid	1.48	1 [Reference]	1 [Reference]

CI, confidence interval.

^a^Incidence rate was calculated as cases per 10 000 person-years.

^b^Adjusted for fentanyl patch usage (maximal dose, total amount, and date of use) and additional surgery.

* statistically significant.

### Subgroup analyses

In subgroup analyses of composite outcome, there was a significant difference in male subgroup (HR 1.26 [1.02–1.55], corrected *P = *0.035), female subgroup (HR 1.19 [1.01–1.40], corrected *P = *0.035), no history of alcohol subgroup (HR 1.20 [1.05–1.39], corrected *P = *0.018), and the no emergency operation subgroup (HR 1.23 [1.08–1.41], corrected *P = *0.004) between the fentanyl and other opioid users (Table 3). In the subgroup analyses of depression, there was a difference in the female subgroup (HR 1.21 [1.00–1.47], *P = *0.094) and the no emergency operation subgroup (HR 1.17 [1.01–1.37], *P = *0.098) between fentanyl and other opioid users, but it was not statistically significant after FDR correction (Supplementary S1 Table). Similar to the main analysis, the risks of anxiety disorder, stress-related disorder, substance use disorder, and psychotic disorder were not significantly different between the fentanyl and other opioid user groups in all subgroup analyses.

**Table 3:** Subgroup analyses of the association between exposure to fentanyl and risk of composite outcome.

	Composite outcome		
Subgroup	Incidence Rate^a^ (Fentanyl/ Other Opioid)	Hazard ratio (95% CI) ^b^	*Corrected P*-value^c^
Sex			
Male	89.60/62.43	1.26 [1.02–1.55]^*^	0.035
Female	115.22/94.90	1.19 [1.01–1.40]^*^	0.035
Alcohol history			
Yes	84.32/63.34	1.29 [0.94–1.58]	0.114
No	107.38/84.09	1.20 [1.05–1.39]^*^	0.018
Emergency operation			
Yes	135.99/127.16	1.01 [0.64–1.61]	0.951
No	100.76/77.10	1.23 [1.08–1.41]^*^	0.004
Operation type			
Neurosurgery	51.46/51.08	1.01 [0.51–2.03]	0.968
Thoracic surgery	195.31/189.08	1.02 [0.53–1.95]	0.968
Head and neck surgery	166.72/164.89	1.02 [0.69–1.51]	0.968
Breast/endoscopic surgery	137.63/139.10	0.98 [0.68–1.43]	0.968
Stomach surgery	71.09/43.75	1.48 [0.98–2.26]	0.215
Hepatobiliary surgery	78.84/44.35	1.81 [1.03–3.17]	0.198
Colorectal surgery	100.81/98.32	0.98 [0.69–1.39]	0.968
Urogenital surgery	79.76/71.89	1.13 [0.76–1.69]	0.968
Gynecological surgery	91.85/80.62	1.05 [0.54–2.06]	0.968
Orthopedic surgery	111.08/77.39	1.30 [1.01–1.67]	0.198

CI, confidence interval.

^a^Incidence rate was calculated as cases per 10 000 person-years.

^b^Adjusted hazard ratio for fentanyl patch usage (maximal dose, total amount, and date of use) and additional surgery.

^c^P-values significant after Benjamini–Hochberg false discovery rate correction for multiple comparisons at a 0.05 threshold.

* statistically significant.

## Discussion

In this retrospective study, our findings indicate a potential association between fentanyl exposure during perioperative care and an increased risk of psychiatric disorders compared to other opioids. This observation contributes valuable insights to our understanding of the potential long-term effects of fentanyl analogs in surgical patients. These results highlight the importance of considering not only immediate perioperative outcomes but also potential psychiatric sequelae associated with opioid exposure. Our results underscore the necessity not only for vigilant monitoring of fentanyl analogs to mitigate immediate complications, but also for sustained attention to their potential long-term impact on mental health.

Opioids have revolutionized pain management, ushering in innovative approaches. Although their use is not without potential complications, opioid prescription varies widely, frequently in excess. The rates of postoperative opioid prescription in the United States and Canada were estimated to be seven-fold higher compared with that in Sweden [17]. In addition to well-documented immediate complications, recent attention has shifted toward potential long-term complications. As opioids are known to interact with the central nervous system, questions have arisen regarding their impact on neuropsychiatric aspects, including mood regulation [18]. In numerous studies, chronic opioid exposure has been associated with alterations in neurotransmitter systems and neural plasticity, contributing to an increased vulnerability to psychiatric disorders such as depression and anxiety [19–21]. Our findings extend these observations to the perioperative setting, suggesting that even short-term exposure to high-potency opioids may have lasting effects. Understanding these neurobiological impacts becomes crucial for a comprehensive evaluation of opioid use, encompassing not only the well-known immediate complications, but also the subtler, enduring effects on the body’s neurological framework.

Opioids, including fentanyl analogs, interact with the central nervous system, influencing neurotransmitter systems and neural circuits. This neurobiological impact has been associated with alterations in mood regulation and emotional processing, potentially contributing to the development of psychiatric disorders. Moreover, the psychosocial aspects of opioid use, such as the risk of dependence and the consequences of chronic pain, may further contribute to the observed association with psychiatric disorders. Individuals exposed to opioids, especially over an extended period, may experience changes in social functioning, coping mechanisms, and overall quality of life, influencing mental health outcomes. These findings are aligned with biopsychosocial models of psychiatric vulnerability, emphasizing the interaction of biological susceptibility, perioperative stress, and psychosocial factors.

Fentanyl analogs are specialized for acute pain control due to characteristics such as rapid onset and low cost [6]. However, given the narrow safety margin associated with these features, there is a heightened awareness of increased risks, including a notable prevalence of addiction, reaching endemic levels, and an elevated risk of mortality [9]. The narrow safety margin suggests the potential for a higher incidence of complications [5]. From this perspective, our study compared the long-term psychiatric risks between fentanyl and other opioids. The risk of composite outcomes, defined as a composite of depression, anxiety disorder, stress-related disorder, substance use disorder, and psychotic disorder, was significantly higher in fentanyl users compared to other opioid users. Moreover, the risk of depression was significantly higher in fentanyl users compared with other opioid users. A previous study using Mendelian randomization has suggested that opioid use itself increases the risk of depression [22]. Considering fentanyl is known for its rewarding properties, which significantly contribute to its high potential for abuse [23], fentanyl may have had a greater impact on psychiatric disorders, particularly depression, than other opioids. This reinforces the need for cautious perioperative use of fentanyl analogues, particularly in populations at higher psychiatric risk. Nevertheless, it remains unknown whether the use of fentanyl analogs during appropriate periods, such as the perioperative period, might lead to an increased risk of long-term complications compared to other opioids. As we navigate the complexities of perioperative pain management, it becomes imperative to strike a balance between the analgesic efficacy of fentanyl analogs and potential risks to ensure the overall well-being of surgical patients.

In our subgroup analysis, the risk of composite outcomes in the no history of alcohol subgroup and the no emergency surgery subgroup was significantly higher in fentanyl users. The higher risk associated with fentanyl use in elective surgeries appears to be related to the type of surgery performed. Specifically, hepatobiliary or orthopedic surgeries, which are common in elective settings, showed increased risks with fentanyl use in our findings. In alignment with our study, previous research has also indicated that the risk varies depending on the type of surgery. Alcohol history is typically associated with various mental disorders, including opioid use disorder [24]. While the presence of alcohol history did not show significance, there was a tendency for the composite outcome to increase in our findings. The higher risk associated with fentanyl use in patients with no history of alcohol appears to be related to the female subgroup. Considering that women are much less likely to have alcohol-related issues compared to men [25], the differences in composite outcomes and depression observed in the female subgroup could explain the results in the subgroup with no history of alcohol. These subgroup findings point to the potential value of targeted postoperative psychiatric surveillance in selected patient populations.

The mechanism relating opioid use and psychiatric disease remains unclear, but a possible explanation may involve a complex interplay of neurobiological and psychosocial factors [18]. While our study provides insight into the potential link between fentanyl analog use and psychiatric disorders, further research is essential to unravel the specific mechanisms involved and differentiate the contributions of neurobiological and psychosocial factors. Future studies, including prospective multicenter cohorts and mechanistic investigations, will be critical to define dose–response relationships and identify modifiable risk factors. This understanding will play a pivotal role in developing targeted interventions and refining perioperative pain management strategies to safeguard both immediate and long-term patient well-being.

Our study had several limitations. First, due to the retrospective nature of the study, confounding factors that were not accounted for in this analysis may exist, and causality cannot be established. Second, we conducted this study at a single center, which may limit the generalizability of our findings to patients with different ethnicities or perioperative management. Third, our results do not clearly explain underlying mechanisms between fentanyl use and psychiatric disorder development. In addition, we could also not propose any modality to prevent psychiatric disorder development after fentanyl use in surgical patients or to provide a safe level of dosing. These issues may need to be investigated in further studies.

## Conclusion

In conclusion, fentanyl use during the perioperative period was associated with increased risk of psychiatric disorder compared with other opioids in long-term follow-up. Further prospective studies should be conducted regarding underlying mechanisms and safety of perioperative fentanyl use.

## Supporting information

S1 Table Subgroup analyses of the Association Between Exposure to Fentanyl and Risk of psychiatric disorders. (DOCX)
