# Supplementary material for: Association of perioperative fentanyl analog exposure with risk of psychiatric disorder in non-cardiac surgery: A 10-year retrospective study in a Korean tertiary hospital
Source: PLoS One. 2025 Dec 31;20(12):e0338927. doi: 10.1371/journal.pone.0338927 (PMC12755743; doi:10.1371/journal.pone.0338927)
Supplement: S1 Table — (DOCX) [file pone.0338927.s001.docx]

**Association of Perioperative Fentanyl Analog Exposure With Risk of Psychiatric Disorder in Non-cardiac Surgery: a 10-year retrospective study in a Korean tertiary hospita**

**Supplementary Table 1. Subgroup analyses of the Association Between Exposure to Fentanyl and Risk of psychiatric disorders**

|  | **Depression** | | **Anxiety disorder** | | **Stress-related disorder** | | **Substance use disorder** | | **Psychotic disorder** | |
| --- | --- | --- | --- | --- | --- | --- | --- | --- | --- | --- |
| **Subgroup** | **Incidence Rate^a^**  **(Fentanyl /**  **Other Opioid)** | **Hazard ratio (95% CI) ^b^** | **Incidence Rate^a^**  **(Fentanyl / Other Opioid)** | **Hazard ratio (95% CI) ^b^** | **Incidence Rate^a^**  **(Fentanyl / Other Opioid)** | **Hazard ratio (95% CI) ^b^** | **Incidence Rate^a^**  **(Fentanyl / Other Opioid)** | **Hazard ratio (95% CI) ^b^** | **Incidence Rate^a^**  **(Fentanyl / Other Opioid)** | **Hazard ratio (95% CI) ^b^** |
| Sex |  |  |  |  |  |  |  |  |  |  |
| Male | 58.54/44.69 | 1.12 [0.87 – 1.44] | 31.68/25.88 | 1.11 [0.79 – 1.55] | 14.65/6.75 | 1.74 [0.97 – 3.10] | 4.62/3.17 | 1.40 [0.57 – 3.46] | 2.69/1.19 | 1.85 [0.47 – 7.30] |
| Female | 85.31/69.01 | 1.21 [1.00 – 1.47]* | 39.24/32.59 | 1.19 [0.89 – 1.57] | 14.84/13.13 | 1.14 [0.73 – 1.78] | 1.44/1.72 | 0.82 [0.22 – 3.05] | 1.81/1.72 | 1.06 [0.31 – 3.67] |
| Alcohol history |  |  |  |  |  |  |  |  |  |  |
| Yes | 56.58/45.37 | 1.19 [0.82 – 1.74] | 30.99/24.84 | 1.22 [0.74 – 2.03] | 10.30/8.85 | 1.04 [0.44 – 2.48] | 5.61/2.65 | 2.17 [0.46 – 8.68] | 1.87/0.88 | 2.15 [0.19 – 2.37] |
| No | 76.23/60.92 | 117[0.99 – 1.38] | 36.72/30.69 | 1.15 [0.91 – 1.45] | 15.85/10.51 | 1.44 [0.98 – 2.11] | 2.33/2.33 | 0.91 [0.38 – 2.21] | 2.33/1.63 | 1.32 [0.50 – 3.50] |
| Emergency operation |  |  |  |  |  |  |  |  |  |  |
| Yes | 92.19/73.50 | 1.23 [0.69 – 2.19] | 55.84/59.48 | 0.90 [0.45 – 1.80] | 26.16/13.89 | 1.62 [0.48 – 5.45] | 6.52/3.46 | 1.99 [0.18 – 2.19] | 6.51/0.00 | NA |
| No | 71.10/56.78 | 1.17 [1.01 – 1.37]* | 34.35/27.79 | 1.18 [0.95 – 1.48] | 14.05/9.95 | 1.33 [0.92 – 1.91] | 2.77/2.34 | 1.13 [0.52 – 2.45] | 1.98/1.56 | 1.21 [0.47 – 3.08] |
| Operation type |  |  |  |  |  |  |  |  |  |  |
| Neurosurgery | 20.48/36.77 | 0.57 [0.21 – 1.52] | 30.74/25.44 | 1.17 [0.46 – 2.94] | 13.62/5.62 | 2.34 [0.43 – 12.81] | 0.00/2.81 | NA | 0.00/0.00 | NA |
| Thoracic surgery | 151.27/108.78 | 1.38 [0.60 – 3.13] | 70.27/77.52 | 0.92 [0.33 – 2.54] | 37.16/30.62 | 1.29 [0.27 – 6.14] | 13.91/0.00 | NA | 9.26/0.00 | NA |
| Head and neck surgery | 132.08/112.42 | 1.20 [0.76 – 1.89] | 54.86/61.01 | 0.86 [0.44 – 1.67] | 18.16/23.63 | 0.71 [0.23 – 2.20] | 3.02/10.09 | 0.31 [0.03 – 3.02] | 0.00/10.09 | NA |
| Breast/endo surgery | 109.48/100.08 | 1.09 [0.71 – 1.67] | 49.53/53.66 | 0.90 [0.49 – 1.65] | 22.45/21.29 | 1.04 [0.41 – 2.65] | 0.00/0.00 | NA | 2.23/2.65 | 0.86 [0.05 – 13.81] |
| Stomach surgery | 45.91/36.78 | 1.13 [0.70 – 1.84] | 23.52/11.44 | 1.75 [0.79 – 3.86] | 9.13/5.72 | 1.47 [0.46 –4.64] | 2.60/0.00 | NA | 1.30/0.00 | NA |
| Hepatobiliary surgery | 51.56/31.00 | 1.77 [0.89 – 3.51] | 22.02/19.89 | 1.11 [0.44 – 2.81] | 9.77/2.21 | 4.38 [0.48 –39.71] | 4.88/0.00 | NA | 0.00/0.00 | NA |
| Colorectal surgery | 63.01/67.74 | 0.90 [0.58 – 1.40] | 28.92/29.91 | 0.92 [0.48 – 1.74] | 22.48/17.91 | 1.16 [0.53 – 2.50] | 1.60/2.98 | 0.46 [0.40 – 4.92] | 3.20/0.00 | NA |
| Urogenital surgery | 51.34/53.74 | 0.95 [0.59 – 1.53] | 32.62/29.77 | 1.09 [0.59 – 2.02] | 9.28/5.94 | 2.20 [0.54 – 9.03] | 3.09/4.45 | 0.62 [0.10 – 3.89] | 3.09/1.48 | 2.12 [0.19 – 22.38] |
| Gynecological surgery | 67.44/50.11 | 1.25 [0.55 – 2.83] | 23.91/24.97 | 0.95 [0.27 – 3.27] | 23.91/24.97 | 0.74 [0.20 – 2.64] | 0.00/0.00 | NA | 0.00/4.98 | NA |
| Orthopaedic surgery | 80.54/57.68 | 1.23 [0.92 – 1.65] | 38.94/29.79 | 1.24 [0.82 – 1.87] | 10.57/6.21 | 1.54 [0.67 – 3.57] | 3.52/2.76 | 1.31 [0.35 – 4.89] | 2.82/1.38 | 2.18 [0.40 – 11.93] |

Abbreviations: CI, confidence interval

^a^Incidence rate was calculated as case per 10 000 person-years

^b^Adjusted hazard ratio for fentanyl patch usage (maximal dose, total amount, and date of use)

^*^statistically significant
